# Introduction to special issue on institutional influences on retirement, health and well-being

**DOI:** 10.1017/s1474747221000408

**Published:** 2021-11-22

**Authors:** David Knapp, Jinkook Lee

**Affiliations:** 1 Center for Economic and Social Research, University of Southern California, Washington, DC, USA; 2 Center for Economic and Social Research, University of Southern California, Los Angeles, CA, USA

**Keywords:** retirement, health, well being, Social Security, J26, I18, I38, H55

## Abstract

Countries make differing policy choices. They can serve as a scientific laboratory for drawing lessons on the policy paths to follow or to avoid and the consequences of those institutional choices on individuals at older ages. In this special issue we bring together six articles that evaluate the influence of institutions on retirement decisions, health and well-being of older adults using common data that have emerged with the international network of health and retirement studies to study key life outcomes such as health, work, and lifecycle transitions at older ages.

Policy makers across the world, including the United States, Europe, and Asia, have differentially responded to population aging by adapting their country’s institutions to balance the needs of aging populations with the resources required to support their institutions. Given the different policy choices, countries can serve as a scientific laboratory for drawing lessons on the policy paths to follow or to avoid and the consequences of those institutional choices on individuals at older ages, which we define here as over age 50. Common data to study key life outcomes such as health, work, and lifecycle transitions at older ages have emerged with the Health and Retirement Study (HRS), first developed in the United States but now administered in comparable forms in more than 40 countries on five continents. In this special issue, we bring together research that evaluates the influence of institutions on retirement decisions, health, and well-being of older adults.

Several authors have highlighted relationships between policy and retirement, health, and well-being using international comparisons. The foundational studies documented in Gruber and Wise (1999, 2004) revealed a stark connection between the institutional design of public pensions and labor force exit at older ages. Collectively, these studies revealed that retirement was strongly connected to pension eligibility ages and that the incentives to delay claiming were also associated with delayed retirement. In the following two decades, further research on institutional influences has revealed connections between policies and outcomes. For example, Banks and Mazzonna (2012) use a regression discontinuity design to identify that longer schooling periods lead to improved memory and executive function. Using this same policy variation, Brunello et al. (2016) connect longer required schooling periods to better self-reported health by identifying the role of mediating effects, such as reductions in smoking and drinking or greater exercise, in explaining the education and late-life health relationship. Avendano et al. (2015), using a difference-in-differences design, connect more generous maternal leave policies to lower rates of depression in old age.

This special issue adds to this literature by bringing together papers that leverage the HRS and its international network of studies (HRS-INS) to examine how institutions influence retirement, health, or well-being. We have collected six original papers and arranged them in roughly this order. The first two investigate how retirement incentives in public pensions have shaped labor force participation for urban and rural workers in China and Mexican immigrants in the United States. The second two examine whether institutional policies aimed at supporting those in poor health, either through lower copays for health insurance or early access to old-age pensions for groups that are disabled or in poor health, are effective as designed. The final two examine the consequence of institutional design on the financial well-being of individuals. In the next section, we highlight the motivation, approach, and key findings of each paper.

## 1. Tour through the special issue

Gruber and Wise (1999) highlighted the connection between old-age pension eligibility and retirement in the United States, Canada, Europe, and Japan. In this spirit, Giles et al. (2021) investigate whether China, which has separate systems for urban and rural workers, also reflects this same level of sensitivity to pension design. China offers an interesting alternative given its different political, social, and economic system. The authors highlight how separate old-age systems, one designed for urban workers and the other for rural workers, are associated with starkly different retirement timing. The system for urban workers provides full benefits at age 60 for men, 55 for women in white-collar occupations, and age 50 for women in blue-collar occupations. Using data from the China Health and Retirement Longitudinal Study (CHARLS), a nationally representative survey, the authors find that 71% of individuals living in urban areas are retired between ages 60 and 64, compared to only 26% in rural areas. Controlling for age, education, health, and other key family and demographic characteristics, the authors find that the receipt of the urban pension is associated with 43% and 78% decreases in labor supply for men and women respectively. Given China’s rapidly aging population, urban/rural disparities in old-age work, and growing pension obligations, the authors suggest that there is ample room to leverage pension policy to reduce old-age poverty across the country through pension design.

Another aspect explored by Giles et al. (2021) is the interdependence of household decisions. Consistent with earlier research (e.g., Gustman and Steinmeier, 2004; Blau, 1998), they find a very strong, positive interrelationship between spousal labor supply. If intrahousehold decisionmaking plays an important role in determining retirement timing, then policy design pertaining to collective household benefits may provide a lever for influencing husbands and wives’ decisions in old age, including work but also plausibly extending to other life-cycle decisions such as child, parental, and own care decisions and decumulation of savings in retirement.

Not only across countries, but also within country, substantial variations in means and opportunities exist by gender, ethnic, or racial groups. Aguila et al. (2021) illustrate that harmonized surveys can be leveraged to demonstrate both within and cross-country differences in retirement behaviors for two tightly connected countries: the United States and Mexico. In the US, Mexican-born immigrants have less education, earn less, and have less accumulated wealth than their non-Hispanic white counter-parts. Further, they are substantially less likely to have access to employer pensions compared to non-Hispanic whites (71% versus 34%). Given their low readiness for retirement, the authors explore whether Mexican-born immigrants in the United States respond differently to typical retirement determinants in the US, such as access to health insurance, education level, or incentives to delay Social Security. They find that most typical determinants are not significantly associated with retirement decisions of Mexican-born immigrants, except that they respond similarly to incentives in Social Security. Repeating their analysis using the Mexican Health and Aging Study, they find non-migrant older Mexicans respond to typical health and income determinants (i.e., a greater probability of retirement is associated with poorer health, greater income, access to a public pension), but that retirement of return migrants to Mexico is not responsive to these typical determinants. However, both non-migrants and return migrants in Mexico exhibit different responsiveness to family and demographic characteristics than either group in United States. Namely, men are substantially and significantly less likely to retire than women (there was no relationship in the United States) and having more people in a household is associated with a great likelihood in retirement (the relationship is opposite in the United States for non-Hispanic whites and there is no clear relationship for Mexican-born immigrants). Aguila et al. (2021) speculate that the greater likelihood of retirement in larger households reflects family transfers and pooled resources as a means of supporting exit from the labor force. Future research could help explore how local culture and traditions influence the sensitivity of responses to policy design in old age. The HRS-INS includes a number of countries that have traditional emphasis on filial support, including Mexico, China, Malaysia, Italy, Spain, and Korea, and a number of countries where laws exist but enforcement is limited (e.g., United States) or are being phased out (e.g., in 2018, Austria abolished a requirement for adult children to be financially responsible for long-term care costs of their parents).

A line of research, much of which has been featured in this journal, has demonstrated the sensitivity of people’s decisionmaking to simple behavioral barriers that preempt action, such as default investment choices in retirement accounts. This theme carries over to health insurance and health care utilization through copayments as a means of deterring overuse or a means test for access to government-provided health insurance. The creation of barriers in health insurance are often aimed at deterring unnecessary use, but they can have the side effect of also deterring necessary use among persons that may be particularly sensitive to those barriers. It is possible, for example, that financially illiterate individuals would be less likely to begin an application process for means-tested health insurance. These same individuals, should they have a negative health shock, might then be less likely to seek health care because they lack health insurance or delay seeking care until the health issue becomes more acute. The significance of striking this balance and the need for research on appropriate design to accomplish that balance are highlighted in Yuda and Lee (2021) and Börsch-Supan et al. (2021).

Yuda and Lee (2021) document differences in health and health care utilization in Japan based on an age-related threshold for medical services copayments. Their study highlights the value of combining rich panel data with geographic variation and independent contextual events. The 2011 Great East Japan Earthquake led to an unexpected increase in health care need in localities affected by the earthquake. The universal Japanese health insurance system reduces copayments from 30% to 10% for adults age 70 and older. Using panel data from the Japanese Study of Aging and Retirement before and after the earthquake in both areas affected and not affected by the earthquake, they find significantly better health outcomes for persons age 70 and older in the earthquake-affected areas compared to those under age 70 and thus facing higher copayments. Although they find that a discontinuous decline in the copayment rate for medical care at age 70 had a protective impact on health, it is important to note that the reduction in copayment did not increase health care utilization among those who were affected by the earthquake, suggesting a substitution effect. This relationship is likely not unique to Japan. Yuda and Lee’s findings suggest that public insurance policy design should give greater consideration to emergency contexts, such as natural disasters and sudden and wide-spreading COVID-19 pandemic, and potentially integrate triggers for relaxing barriers to coverage or use against natural disasters, disease pandemic, or other urgent and disasterous circumstances.

Institutions designed to provide a sense of security regarding income and health in old age typically rely on tax revenues or government borrowing, and it is ultimately a country’s current or future population that bears the costs of these institutions. These institutions’ programs are often initially setup to target particular population groups that are perceived to be at greater risk of insecurity or eventually reformed to target limited resources. Example abound including the progressivity of the Social Security benefit in the Unites States, the introduction of the flat basic pensions in the United Kingdom and South Korea, and the introduction of an early eligibility option for women with long work histories and minimum pension supplements for persons over age 70 in Italy. However, coarse eligibility criteria or poor policy design will often lead to outcomes that differ substantively from the original motivation for the targeting. Evaluating policy design is critical to efficient use of limited resources. In this spirit, Börsch-Supan et al. (2021) examine three policy reforms that were intended to provide targeted support to particular groups. These include a reform intended to better target disability pensions to those experiencing a work-related disability, a reform aimed at providing old-age pension benefits at younger ages for workers in arduous jobs with long careers, and a supplemental pension aimed at households with a high poverty risk. Their main finding is that none of these policies do a particularly good job of providing benefits to the intended populations. In the case of the early pension for those in arduous jobs, the authors find the eligible beneficiaries have neither lower income or poorer health than otherwise similar but ineligible individuals. As a result, this reform has just created another alternative pathway to benefit eligibility that encourages earlier exit from the labor force. Regarding the supplemental pension, the authors highlight that the service year requirement precludes much of the target population and the lack of an asset test allows many wealthy recipients to receive the additional benefit.

One of the main aims of social policy is to insure the well-being of individuals in a society. Countries vary in the level of social insurance and assistance they provide to individuals in old age, but the aim is typically the same – provide a minimal level of support for individuals as they enter the later stages of life when physical, health, and cognitive changes can impose limits on ability to regularly work and generate income. Government old-age support is typically provided in the form of income and in-kind medical services. Income poverty is a metric by which financial well-being is most often assessed and the aim of many countries is to design their old-age social insurance and assistance systems to reduce the likelihood of individuals falling into poverty after they exit the labor force or to escape poverty as they enter the later stages of life.

This is where El-Attar Vilalta and Fonseca (2021) pick up their research – they ask whether the Canadian social assistance system, consisting of multiple means-tested programs, is effective at preventing old-age individuals from falling into poverty or in lifting them out of it. Using OECD’s measure of poverty – having an income less than one-half of a country’s adjusted median household income – Canada’s poverty rate is in the middle of high-income countries (OECD, 2019). Critical to their analysis is understanding the dynamics of poverty. Consistent with past research (Valetta, 2006; Finnie et al., 2013; Hansen et al., 2014), they find that poverty in Canada is highly persistent. In terms of means-tested old-age social assistance, Canada has three benefit systems: Old Age Security, Guaranteed Income Plan, and the Allowance, all of which are only income tested. These are distinct from the non-means-tested, contributory old-age social insurance program known as Canada/Quebec Pension Plan. To analyze the impact of the social assistance programs on poverty, the authors use person-level data from three survey waves (2012–2016) of the Canadian Longitudinal and International Study of Adults which measures income, health status, and other socio-demographic factors. The survey data are linked to administrative data that track individual’s earnings from 2001 that also include a measure of relative poverty. While no natural experiment exists during this time period, El-Attar Vilalta and Fonseca (2021) leverage individual variation in eligibility for these programs due to income and circumstance (e.g., individuals between age 60 and 64 are eligible for the Allowance if their spouse is receiving the Guaranteed Income Supplement) to identify a relationship between eligibility for these programs and poverty. They consistently find across their analysis involving different models and specifications that receiving Canadian social assistance is associated with significant and meaningful improvements in the probability of exiting poverty. They also find it is not consistent with a reduced probability of entry into poverty, although they find receipt of the Canadian social insurance benefit is associated with a lower probability of entry into poverty. Taken together, El-Attar Vilalta and Fonseca’s (2021) analysis suggests that poverty is highly persistent across the life-cycle and social assistance programs can help the marginally impoverished escape poverty but that even with this support it does not eliminate poverty in old age. They propose that reducing poverty in old age may require more focused efforts earlier in the life-cycle so that initial entry into poverty does not become a persistent state. We expect the limitations for alleviating poverty highlighted by El-Attar Vilalta and Fonseca (2021) of the Canadian old-age social assistance programs are likely to be shared by similar programs in other high-income countries. Documenting the role of poverty persistence in limiting the effectiveness of old-age social assistance programs and identifying potential policy mechanisms for preventing individuals from entering poverty across the lifecycle are key parts of the research agenda for improving the well-being of financially fragile persons as they enter old age.

In the United States, the social insurance system is the dominant form of old-age income support, owing to the maturity of the system and broad coverage, but also the stringent income and asset tests associated with the old-age social assistance system. Old Age and Survivors Insurance, colloquially referred to as Social Security, provides strong financial incentives to delay claiming from the earliest eligibility age, 62, to later ages. If a person born between 1943 and 1954 delayed claiming from age 62 to 70 without continuing work, they would increase their monthly benefit by 76%. Despite these incentives, a large majority of beneficiaries still claim at age 62. There has been significant research highlighting the benefits and feasibility of delayed claiming for ensuring the retirement security of beneficiaries (Shoven and Slavov, 2014; Goda et al., 2018). But there has been little evidence on how early claimants fare after claiming. Armour and Knapp (2021) ask whether claiming at age 62 has discernable differences in financial and other well-being outcomes. They exploit the long panel of the HRS to match individuals who do and do not claim at age 62 but are otherwise observationally similar at age 60 along a number of dimensions, including demographics, whether they are working, and their income, assets, health, and survival expectations. Using their matched group of men who are married and working at age 60, they estimate different outcomes across their 60s and 70s. Armour and Knapp (2021) find that the consequences of claiming at the earliest eligibility age are as expected: greater social security income after age 64, greater likelihood of continuing work, and further accumulation of assets for those that delay claiming. Importantly, they find no difference in mortality or measures of financial hardship. They conclude that the difference in financial well-being at later ages between age 62 claimants and those that delay can largely be attributed to differences in these groups that preceded the claiming decision itself. Many countries, including the United Kingdom, Italy, and Korea have implemented or increased incentives to delay benefit claiming in the past 20 years. Armour and Knapp’s work suggests that future research could examine whether efforts to promote delayed claiming have measurable savings for the Social Security systems, benefits for recipients, and the role they play in limiting or increasing financial disparities in old age.

## 2. Future paths

The six papers in this special issue offer a sampling of the possibilities of what can be learned through the combination of harmonized panel survey data, linked administrative data, and detailed knowledge of the evolution of policy design and local context. Rich data and detailed information on policy change are the launchpad for research into the influence of institutional design on retirement, health, and well-being. The HRS-INS continues to expand, adding Malaysia, Chile, and Brazil in recent years and now includes 47 countries. Many of these surveys are now conducting life history surveys that can provide a retrospective view of lifecycle work, health, and relationship outcomes. The Gateway to Global Aging Data (https://g2aging.org) continues to expand harmonized data to facilitate cross-national comparisons and support data access. Further, the Gateway has created the Institutional Rules Explorer Series (https://g2aging.org/institutional-rules) that tracks the evolution of public pension policy since 1992 in HRS-INS countries and will expand in the coming years to include long-term care policies and policy responses to the COVID-19 pandemic. Using detailed policy data, the Gateway is also constructing harmonized measures of pension and long-term care eligibility for each individual at each survey wave and prospective measures of future eligibility that can be easily added to the analyses of retirement, care giving or receiving, or other health outcomes.

As countries make different policy choices, comparing their approaches and outcomes offer insight to researchers, policymakers, and other stakeholders into paths to follow or to avoid and the consequences of those institutional choices on individuals. Overcoming high startup costs for technically complex areas of research, the Gateway and HRS-INS are placing greater emphasis on enabling researchers to jump into their research while incorporating related policy and contextual data that have proven important to health and retirement decisions and outcomes. The Gateway and HRS-INS continue to serve as a critical data source for analyzing the impacts of policy responses to health, retirement, well-being, population aging, and now the global COVID-19 pandemic.
